# Agrivoltaics Combined with Integrated Water–Fertilizer Management Promotes Soybean Yield in a Semi-Arid Sandy Region

**DOI:** 10.3390/life16071062

**Published:** 2026-06-25

**Authors:** Xiaojin Zou, Jiayi Xu, Yiwen Huang, Muyu Tian, Ziqi Liu, Tingting Li, Jiaji Wang, Liang Gong, Liangshan Feng

**Affiliations:** 1 Institute of Plant Nutrition, Resources and Environment, Liaoning Academy of Agricultural Sciences, Shenyang 110161, China; zouxiaojin0501@163.com (X.Z.); x_jy0906@163.com (J.X.); yiwen13940381697@163.com (Y.H.); tian_muyu@163.com (M.T.); wiwix@163.com (Z.L.); gongliang1900@sina.com (L.G.); 2 Liaoning Key Laboratory of Dryland Conservation Tillage, Shenyang 110161, China; 13841894777@163.com; 3 Key Laboratory of Black Soil Cultivation and Fertility Improvement Technology, Ministry of Agriculture and Rural Affairs, Shenyang 110021, China; litingting03@sinochem.com; 4 Zhangwu Hongtai Agriculture and Animal Husbandry Technology Co., Ltd., Fuxin 123200, China

**Keywords:** agrivoltaics, soybean, semi-arid sandy region, light stress, water stress

## Abstract

Horqin Sandy Land suffers from desertification, drought, and low fertility, limiting soybean production. Agrivoltaics provides a promising integrated model; however, the effects of agrivoltaics combined with water–fertilizer management on crop productivity remain unclear. A 2-year field experiment was conducted in a semi-arid area with three treatments, open-field control (Open), shaded area under panels (Under), and light-exposed area inter-panels (Gap). Results showed that photovoltaic systems combined with integrated water–fertilizer management improved soybean yield, soil water, and nutrient conditions. Soybean grain yield was 60.7% and 38.2% higher in the Gap and Under treatments, respectively, than in the Open. The highest yield in the Gap treatment resulted from both enhanced photosynthesis and improved root development. The Under endured light stress but exhibited morphological plasticity (plant height and leaf area increased by 43.1%, 48.2%), and shading alleviated water stress since soil water content was increased by 81.6–119.0% during growing seasons, transpiration rate (*Tr*) decreased by 55.1%, and leaf water use efficiency (WUE) increased by 48.8%. The Open suffered from soil degradation and water and fertilizer loss, resulting in severely limited yield. Agrivoltaics increased net income by 1466 CNY·ha^−1^ and improved soil nutrients, demonstrating economic and ecological benefits. Thus, it is a suitable technical model for semi-arid sandy regions.

## 1. Introduction

Horqin Sandy Land is a typical semi-arid sandy region in northern China, where severe soil desertification, soil compaction, and nutrient depletion have triggered continuous degradation of cultivated land quality [1]. This has become a major bottleneck restricting the improvement of regional agricultural production efficiency and food security. Meanwhile, as a key part of the northern ecological security barrier in China, this region bears the dual tasks of combating desertification and achieving sustainable agricultural development. Conventional farming systems can no longer meet these demands, calling urgently for an integrated agricultural model that combines ecological restoration, efficient resource utilization, and grain production.

Agrivoltaics, an innovative and sustainable dual-land-use model that integrates solar photovoltaic power generation with agricultural production, represents a crucial strategy for alleviating the imbalance between the supply and demand of land resources [2]. It could coordinate clean energy supply with food security, promote the green transformation of the agricultural sector, and support the achievement of national carbon peaking and carbon neutrality goals [3]. In recent years, diversified agrivoltaic systems, such as agrivoltaic complementation, fishery-photovoltaic integration, forest-photovoltaic combination, and photovoltaic ecological restoration, have gained extensive attention and rapid deployment worldwide [4,5,6,7]. Benefiting from abundant solar radiation and a large-scale agricultural production system, China has become one of the countries with the most rapid development and diversified application scenarios of global photovoltaic agriculture [8,9]. This integrated model shows substantial advantages in improving land-use efficiency, diversifying agricultural income, and enhancing ecological conditions [10,11,12]. Accordingly, it represents a promising approach for integrating ecological governance and agricultural production in semi-arid sandy regions [13].

Previous studies in ecologically fragile semi-arid regions have preliminarily explored photovoltaic sand fixation, photovoltaic-assisted degraded grassland improvement, and photovoltaic-supported dryland farming, thereby verifying the feasibility of agrivoltaics in ecological restoration and economic development. Agrivoltaic systems exhibit strong microenvironmental heterogeneity, with distinct differences in light, moisture, and soil conditions between under panel and inter panel areas. These differences directly regulate crop growth, photosynthetic physiology, and yield formation [14,15]. However, most previous studies have primarily focused on superficial indicators, such as the analysis of impacts on the ecological environment and the trade-off between photovoltaic power generation and crop yields [16,17]. However, less attention has been paid to how heterogeneous light–water–soil interactions within photovoltaic arrays jointly regulate crop physiological adaptation and yield formation. The southern Horqin Sandy Land provides a unique field system for investigating these mechanisms, where crop production is not limited by insufficient light, but rather by combined stresses including drought, wind erosion, low soil fertility, and weak water–fertilizer retention capacity.

Soybean, an essential grain and oil crop in China, is one of the main economic crops cultivated in the semi-arid sandy region of the southern Horqin Sandy Land. Global agrivoltaics research indicates that soybean, a C3 crop, is more adaptable to shading agrivoltaics environments than C4 maize, making it more suitable for photovoltaic agricultural models in semi-arid regions [18]. Through multi-crop and multi-variety regional adaptability screening trials, our team identified that soybean cultivar Liaohei No. 5 exhibits strong shade tolerance and resistance to infertile soils under agrivoltaic systems in the semi-arid study area. Accordingly, a two-year field experiment was conducted from 2024 to 2025 using the soybean cultivar Liaohei No. 5 as the test material. The specific objectives of this study were to: (1) evaluate whether agrivoltaic systems combined with water–fertilizer management can improve crop productivity and soil conditions in degraded semi-arid sandy cropland; and (2) determine how light–water–soil heterogeneity induced by photovoltaic arrays regulates soybean root morphology, photosynthetic traits, leaf water-use efficiency, and aboveground morphological adaptation. We hypothesized that photovoltaic arrays would regulate heterogeneous light, water, and soil microenvironments, thereby inducing distinct adaptive strategies of soybean across different microhabitats. The findings are expected to provide a theoretical basis and technical support for the optimized application of agrivoltaics, and sustainable agricultural development in the southern Horqin Sandy Land regions.

## 2. Materials and Methods

### 2.1. Experimental Design and Treatment

The field experiment was conducted in a randomized complete block design with three replicates. The experiment is located in Haertao (42°37′ N, 122°27′ E), Zhangwu Couty, Fuxin City, Liaoning province, China. It is at the southern edge of the Horqin Sandy Land, a typical semi-arid aeolian sandy region, with a multi-year average precipitation of 391 mm in the past 30 years and a mean annual wind speed of 3–4.4 m/s. The number of sand-driving wind days (≥5 m/s) ranges from 200 to 350 days per year. The soil type is sandy loam with a pH of 5.53, organic matter of 4.38 g kg^−1^, total N of 0.63 g kg^−1^, available P of 9.27 mg kg^−1^, and available K of 39.5 mg kg^−1^, within the 0–20 cm soil layer.

### 2.2. Experimental Design

**Figure 1:**
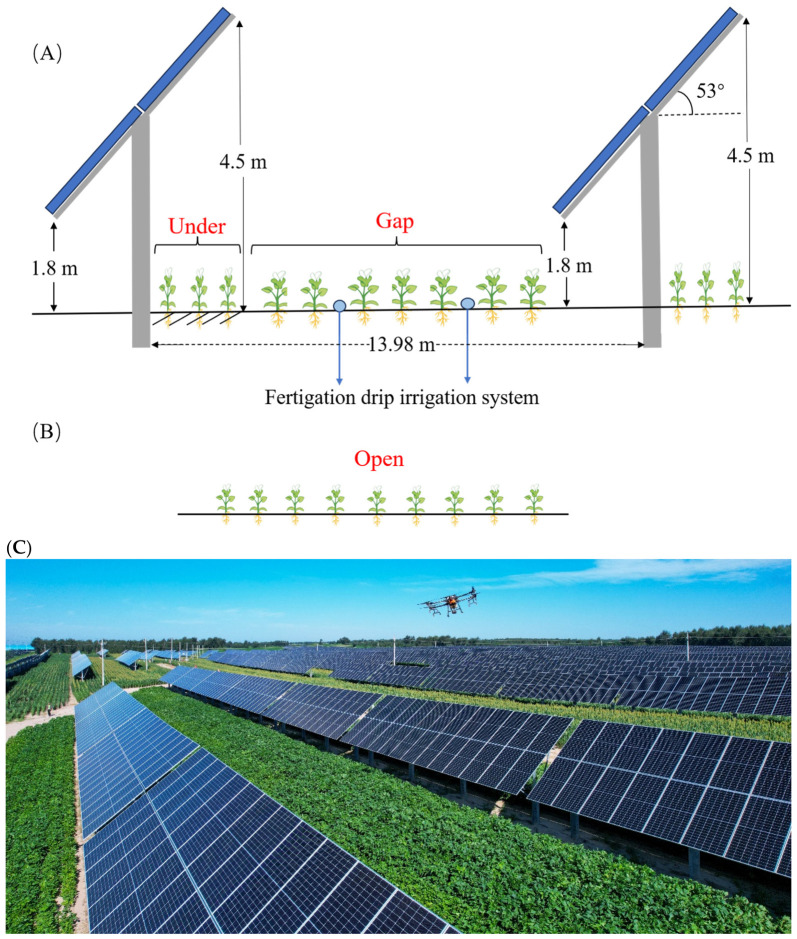
Schematic diagram of photovoltaic panels, (**A**) Under: fully shaded area beneath photovoltaic panels; Gap: between photovoltaic panel arrays with no light stress; (**B**) Open, local conventional open-field cropping without photovoltaic panels or integrated water–fertilizer regulation; (**C**) Agrivoltaic system located in Zhangwu Couty, Fuxin City, China.

The experiment was conducted with a randomized complete block design, involving three treatments and three replications. The plot size was 72 m^2^ (9 m by 8 m). Plots were 1 m apart from each other. The planting layout as well as the parameters of photovoltaic (PV) panels remained consistent across the two years. The three treatments were set as follows: (1) open-field conventional control (Open), which was managed using local traditional planting and cultivation practices; (2) fully shaded area beneath photovoltaic panels (Under); (3) between photovoltaic panel arrays with no light stress (Gap). The photovoltaic panels are monocrystalline silicon modules with an inclination angle of 37°. The height is 1.8 m at the down-slope edge and 4.5 m at the up-slope edge. The space between adjacent photovoltaic modules is 13.98 m. The schematic diagram of the photovoltaic array is shown in Figure 1. The Open treatment represented local conventional open-field cropping without photovoltaic panels or integrated water–fertilizer regulation. It was characterized by severe water stress, marked soil compaction, intense wind–sand erosion, and substantial water and nutrient losses. In contrast, the Gap and Under serve as agrivoltaics, equipped with integrated water and fertilizer facilities for precise water and fertilizer management.

The test cultivar was soybean Liaohei No. 5, a shade-tolerant and barren-tolerant variety which was proved by our group in early-stage experiments. Soybean was sown mechanically on 16 May 2024 and 18 May 2025, and harvested on 10 October 2024 and 8 October 2025, respectively, with a planting density of 210,000 plants·hm^−2^ in both years. Mechanical operations took place throughout the whole soybean growth seasons, including a series of technical measures such as mechanical sowing, organic fertilizer application, subsurface drip irrigation combined with micro-sprinkler irrigation, unmanned aerial vehicle (UAV) aerial pest control, and mechanical harvesting. At sowing, 900 kg·hm^−2^ of inorganic compound fertilizer (N:P:K = 12:18:15) and 30,000 kg·hm^−2^ of organic fertilizer were applied. Irrigation was performed five times per year, with an application rate of 30–40 m^3^ per mu each time depending on soil moisture conditions. Foliar fertilizer (microbial water-soluble preparation from Nanjing Agriculture University) was sprayed three times annually, applied at the seedling, flowering and filling stages of soybean respectively. During the growing period, integrated mechanical and manual weeding, green prevention and control of diseases and insect pests, and other field management measures were carried out as needed. For the Open treatment, the type and application rate of inorganic fertilizers were consistent with those in the agrivoltaics, while without a fertigation drip irrigation system.

### 2.3. Measurements of Photosynthesis, Root Characteristics, Plant Height, Leaf Area, Yield, and Yield Components

The photosynthetic parameters of Liaohei No. 5 soybean, including net photosynthetic rate (*Pn*), stomatal conductance (*Gs*), intercellular carbon dioxide concentration (*Ci*), and transpiration rate (*Tr*), were measured using LI-6400 (Li-Cor, Lincoln, NE, USA) from 9:30 to 11:30 a.m. on a sunny day at the flowering stage in 2024–2025. During measurements, the chamber conditions, including the reference CO_2_ concentration, chamber temperature, and relative humidity, were not artificially standardized, but followed the actual field conditions. The airflow rate was kept constant according to the instrument operating protocol to ensure stable gas exchange readings. Five consecutive Liaohei No. 5 soybean plants were measured in each plot, and the measured leaves were the top fully expanded leaves of the canopy. Each measurement was repeated three times to minimize instrumental errors. All measurements were conducted during the same time window and under clear-sky conditions to minimize diurnal variation and ensure comparability among treatments.

Photosynthetically active radiation (PAR) above the soybean canopy was measured at the V4 stages on sunny days between 09:00 and 12:00 in 2024 and 2025, following the method of Huang et al. [19], using a linear quantum meter AccuPAR LP-80 Ceptometer (Decagon Devices, Pullman, WA, USA). Five readings of PAR were taken from five plants in each plot. Each measurement was repeated three times.

Plant height and leaf area were monitored periodically at the flowering stage. Liaohei No. 5 soybean height was measured at harvest season in both years. At each measurement, five plants were randomly selected per plot. Plant height was measured from the soil surface to the tip of the uppermost fully expanded leaf. Individual leaf area was estimated as leaf length multiplied by leaf width and then by shape factor [20,21].

According to the root sampling method described by Huang et al. [19], the monolith method proved to be more suitable than the auger core method in legumes. For each plot, four soybean root monoliths were collected (monolith size for soybean is 0.6 × 0.6 × 0.6 m). Root samples were rinsed in tap water and adhering soil particles were carefully removed with brushes in the lab. Then, the root samples were scanned using a root-scanning instrument (LAD2400, Sintek, Shanghai, China). Images were analysed using WinRhizo (Sintek) to obtain total root length, diameter and surface area. The data was averaged from 4 root samples per plot.

Grain yield was determined from the central 12 m^2^ area (6 m × 2 m) of each plot. Seed samples were sun-dried to approximately 14% moisture content before weighing. Yield components were investigated from five crops in each plot.

### 2.4. Measurements of Soil Moisture and Nutrients Contents

Soil samples were collected from the 0–20 cm tillage layer of each plot using a soil auger at the seedling, flowering, and grain-filling stages of soybean. Each treatment was sampled in triplicate. Soil water content was determined by oven drying at 105 °C to constant weight.

At harvest, five soil subsamples were collected from the 0–20 cm soil layer of each plot and combined to one sample. The samples were composited, air-dried, and sieved through a 2 mm mesh before analysis. Soil pH was measured using a potentiometric method at a soil-to-water ratio of 1:2.5. Soil organic matter was determined by the external heating potassium dichromate oxidation method. Total nitrogen was analysed using the Kjeldahl method. Total phosphorus was determined by the molybdenum–antimony colorimetric method, and total potassium was measured by flame photometry. Alkali-hydrolyzable nitrogen was determined by the alkaline hydrolysis diffusion method. Available phosphorus was extracted with sodium bicarbonate and measured by the molybdenum-antimony colorimetric method, whereas available potassium was extracted with ammonium acetate and determined by flame photometry. All measurements were performed according to the standard procedures [22].

### 2.5. Calculations

Leaf water use efficiency (WUE) is calculated based on the measured photosynthetic parameters, i.e., the ratio of photosynthetic rate (Pn) to transpiration rate (Tr). The equation was as follows:

Leaf WUE = Pn/Tr

The economic benefits of the agrivoltaic system were calculated as the difference between total revenue and production costs. Production costs included seeds, fertilizers, agricultural machinery operation, fertigation and drip irrigation (including facility investment and application costs), and labor. Total revenue was estimated by multiplying soybean yield by the unit price. Soybean yield in the agrivoltaic system was defined as the weighted average yield of the Gap and Under areas. The photovoltaic panel structure covered 80% of the total land area, of which 20% corresponded to the Under area and 60% to the Gap area. In 2025, the cost of water–fertilizer integration facilities were set to zero because the fixed investment had already been fully amortized.

### 2.6. Statistical Analysis

Normality was evaluated using the Shapiro–Wilk test prior to analysis of variance. A two-way ANOVA was applied to test the effects of year and treatment on the yield and yield components, plant height, leaf area, available PAR, photosynthesis (Pn, Gs, Ci, and Tr) and root morphological traits of soybean, and soil moisture and nutrients. The *p*-values from the two-way analysis of variance are presented in Table 1. For variables without a significant interaction effect, only the main effects were compared, whereas for variables with a significant interaction effect, treatment comparisons were conducted within each year. Analyses were performed using SPSS 25.0 (IBM, New York, NY, USA). When the ANOVA revealed significant treatment effects, mean comparisons were performed using Tukey’s honestly significant difference (HSD) test at *p* < 0.05.

## 3. Results

### 3.1. Soybean Yield, Yield Components and Harvest Index (HI)

Analysis of variance (ANOVA) showed that the pod number per plant, grain number per plant, 100-grain dry weight, straw yield and grain yield of soybean were significantly increased in agrivoltaics system compared with the Open treatment (Table 2). The interaction between treatment and year was only significant for 100-grain dry weight. The results showed that pod number per plant, grain number per plant, and 100-grain dry weight in the Gap and Under were significantly higher than those in Open treatment in 2024–2025. For straw, grain yield, and HI, the Gap was the highest, followed by Under, with the Open treatment showing the lowest. Specifically, compared with the Open treatment, the two-year average yields of straw and grain in the Gap were increased by 36.0% and 60.7%, respectively, while compared with those in the Under, straw and grain yields were increased by 13.0% and 16.3%, respectively. The straw and grain yields in the Under were 20.4% and 38.2% higher than those in the Open treatment.

### 3.2. Plant Height and Leaf Area

**Figure 2:**
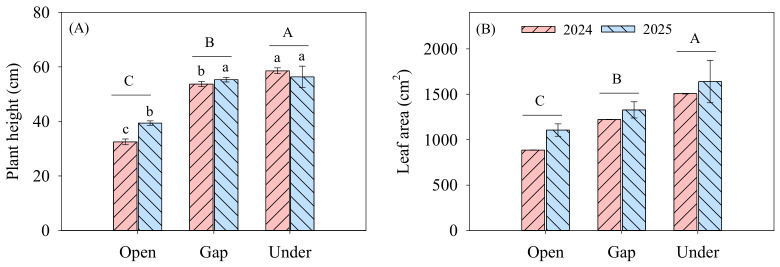
Soybean plant height (**A**) and leaf area (**B**) in the conventional planting and in the agrivoltaics during 2024 and 2025. Open, local conventional open-field cropping without photovoltaic panels or integrated water–fertilizer regulation; Gap, the area between the panels in agrivoltaics treatment; Under, the area under the panels in agrivoltaics treatment. The bars indicate the standard error of triplicates. The different uppercase letters indicate main effects of treatments had significant impacts on plant height and leaf area (*p* < 0.05). The lowercase letters indicate significant differences among different treatments in the same year (*p* < 0.05).

Analysis of variance (ANOVA) suggested that both the cultivation pattern and year had a significant effect on plant height and leaf area (Figure 2). The interaction between cultivation pattern and year indicated a significant influence on plant height, while no significant interaction effect was observed for leaf area. With respect to the main effect of cultivation pattern, the results of plant height and leaf area were as follows: the Under was the highest, followed by the Gap, and the Open was the lowest. Plant height of the Under was increased by 5.44% and 59.8% on average over the two years compared with that of the Gap and Open treatments, respectively. The results indicated that agrivoltaics could significantly promote plant growth, depending on the year to a certain extent. The leaf area of the Under was increased by 23.3% and 58.0% on average when compared with that of the Gap and the Open across the two years, respectively. This suggested that the agrivoltaics system, especially the under-panel treatment, was conducive to leaf expansion and canopy establishment.

### 3.3. Photosynthetically Active Radiation (PAR) and Photosynthetic Parameters

**Figure 3:**
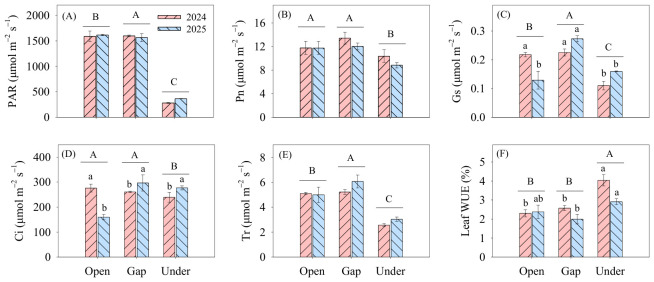
Photosynthetically active radiation (PAR, (**A**)) above the canopy and net photosynthesis rate (Pn, (**B**)), stomatal conductance (Gs, (**C**)), intercellular CO_2_ concentration (Ci, (**D**)), transpiration rate (Tr, (**E**)), and leaf water-use efficiency (WUE, (**F**)) of soybean in the conventional planting and in the agrivoltaics during 2024 and 2025. Open, local conventional open-field cropping without photovoltaic panels or integrated water–fertilizer regulation; Gap, the area between the panels in agrivoltaics treatment; Under, the area under the panels in agrivoltaics treatment. The bars indicate the standard error of triplicates. The different uppercase letters indicate that the main effects of treatments had significant impacts on these indicators (*p* < 0.05). The lowercase letters indicate significant differences among different treatments in the same year (*p* < 0.05).

The PAR in the Gap was significantly higher than that in the Under, where significant light stress was observed (Figure 3). The results showed that the PAR above the soybean canopy in the shaded Under was 78.9% lower than that in the Open treatment. The net photosynthetic rate (*P_n_*) of soybean in the Under was decreased by 18.3% and 24.6% compared with the Open and the Gap, respectively. The transpiration rate (*T_r_*) was decreased by 44.5% and 50.4%, respectively, and the stomatal conductance (*G_s_*) and intercellular CO_2_ concentration (*C_i_*) were also significantly reduced. The *P_n_*, *T_r_* and *G_s_* of soybean in the Gap showed an increasing trend compared with the Open treatment, with a significant increase in *G_s_* and *T_r_* by 43.5%, and 11.8%, respectively. The water-use efficiency (WUE) in the shaded Under was increased by 75.4% and 56.8% in 2024, and by 22.5% and 46.0% in 2025, compared with the Open and Gap treatments, respectively.

### 3.4. Soybean Root Morphology

**Figure 4:**
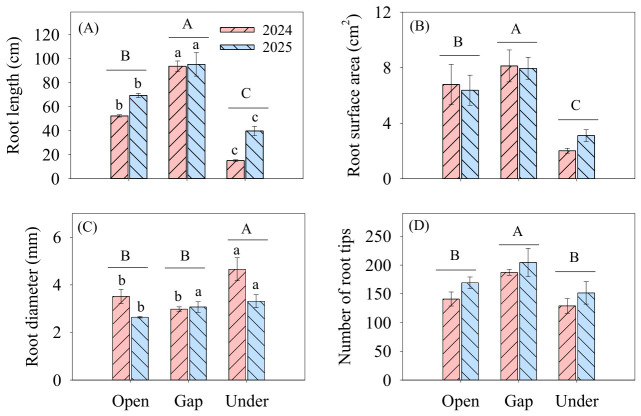
Root length (**A**), surface area (**B**), diameter (**C**), and number of root tips (**D**) in the conventional planting and in the agrivoltaics during 2024 and 2025. Open, local conventional open-field cropping without photovoltaic panels or integrated water–fertilizer regulation; Gap, the area between the panels in agrivoltaics treatment; Under, the area under the panels in agrivoltaics treatment. The bars indicate the standard error of triplicates. The different uppercase letters indicate main effects of treatments had significant impacts on root morphology (*p* < 0.05). The lowercase letters indicate significant differences among different treatments in the same year (*p* < 0.05).

Root surface area and root tip number were increased by 55.2%, 22.0%, and 26.1% in 2024–2025 compared with those in the Open treatment (Figure 4), indicating that the Gap was more conducive to the vertical elongation of roots, the expansion of root absorption interface and the maintenance of strong root meristematic capacity. Root growth in the shaded Under was significantly inhibited by light stress, with the root length, root surface area, and root tip number being only 27.5 cm, 2.57 cm^2^, and 140 on average over two years. These values were decreased by 70.9%, 68.1%, and 28.4% compared with those in the Gap, respectively, while the root diameter was increased by 29.5% compared with the Open treatment. The Gap showed the most obvious advantages in promoting root length increase, root surface area expansion, and root tip formation, whereas the Under was more favorable for root thickening. These results suggested that the agrivoltaics system could significantly regulate the root architecture of Liaohei No. 5 soybean, and the Gap was the most beneficial to the comprehensive root morphological development.

### 3.5. Water Content of Soil

**Figure 5:**
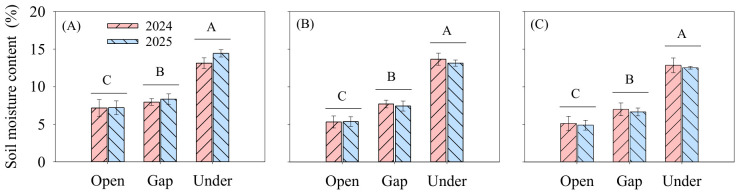
Soil moisture at the seedling stage (**A**), flowering stage (**B**), and filling stage (**C**) of soybean in the conventional planting and in the agrivoltaics during 2024 and 2025. Open, local conventional open-field cropping without photovoltaic panels or integrated water–fertilizer regulation; Gap, the area between the panels in agrivoltaics treatment; Under, the area under the panels in agrivoltaics treatment. The bars indicate the standard error of triplicates. The uppercase letters indicate significant differences among different treatments (*p* < 0.05).

Soil water content at the seedling, flowering, and filling stages was the highest in the shading Under, followed by the Gap, and the lowest in the Open treatment (Figure 5). Compared with conventional planting in the Open treatment, soil water content in the light-sufficient Gap was increased by 13.4%, 41.7%, and 36.3% at the seedling, flowering, and filling stages of Liaohei No. 5 soybean, respectively, while in the shading Under, it increased by 91.7%, 151%, and 153% at the corresponding stages. The agrivoltaics especially the shading Under, maintained a relatively high soil water content throughout the whole growth period, which significantly enhanced the soil water storage and conservation capacity. A more favorable soil water environment was formed with no water stress occurring at the flowering and filling stages, thus providing a stable water supply for soybean grain development.

### 3.6. Soil Nutrient Contents

**Figure 6:**
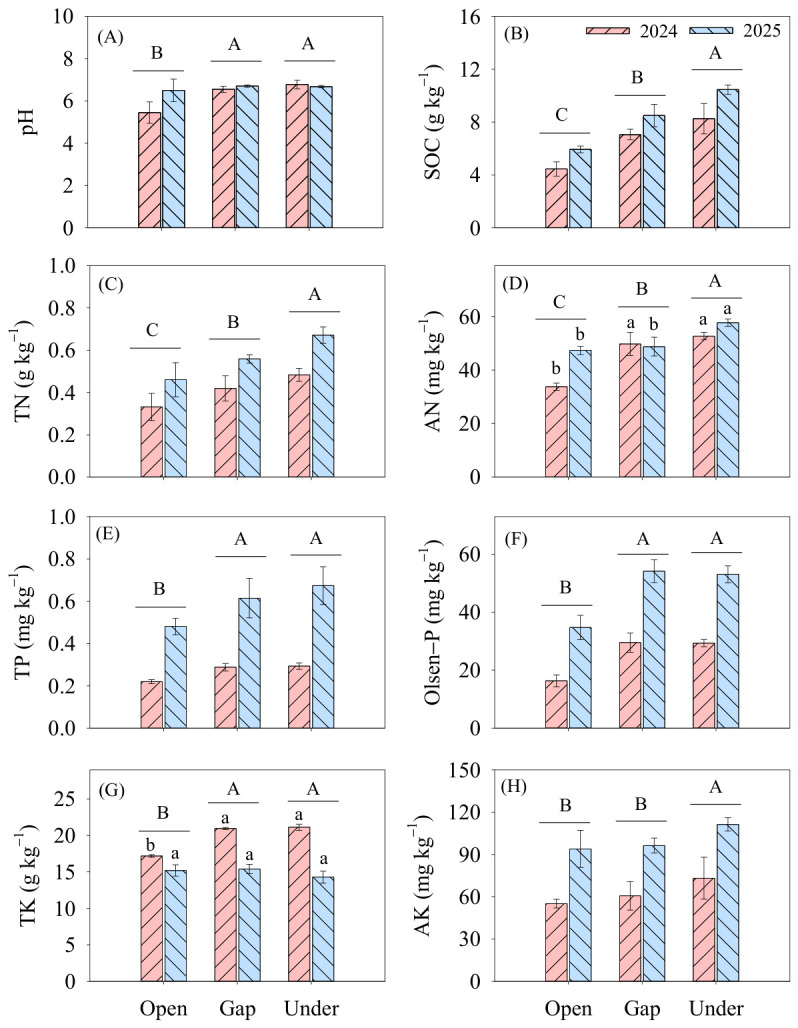
Soil pH (**A**), organic carbon (**B**), and nutrients ((**C**), total nitrogen; (**D**), available nitrogen; (**E**), total phosphorus; (**F**), Olsen-P; (**G**), total potassium; (**H**), available potassium) in the soybean conventional planting and in the agrivoltaics during 2024 and 2025. Open, local conventional open-field cropping without photovoltaic panels or integrated water–fertilizer regulation; Gap, the area between the panels in agrivoltaics treatment; Under, the area under the panels in agrivoltaics treatment. The bars indicate the standard error of triplicates. The different uppercase letters indicate main effects of treatments had significant impacts on soil pH, SOC, and nutrients (*p* < 0.05). The lowercase letters indicate significant differences among different treatments in the same year (*p* < 0.05).

Soil nutrients in the Gap and Under of the photovoltaic array showed an increasing trend both in 2024 and 2025 (Figure 6). Soil organic matter, total nutrients, and available nutrients under photovoltaic arrays were significantly higher than those in the Open treatment. Soil pH in the Gap and Under slightly increased when compared with the Open treatment, while soil organic matter content increased by 49.7% and 80.1%, respectively. In the under-panel zone, the contents of available nitrogen, phosphorus, and potassium significantly increased by 36.0%, 61.5%, and 23.8% compared with the Open treatment, respectively.

### 3.7. Economic Benefits of Agrivoltaics

The economic benefits of the agrivoltaics were slightly higher than those of the Open treatment. The total income per hectare of soybean in the open-field conventional system and the agrivoltaics was 12,226 and 15,161 CNY (Table 3), respectively. Besides the additional costs including mechanical operation, water and fertilizer integration facilities (these facilities can be used 2 or 3 years; the cost of first year is 450 CNY per ha, which is 225 CNY for each year on average), and fertilizers, the net income reached 9582 CNY on average in 2 years per hectare, equivalent to 639 CNY per mu, respectively. Although the agrivoltaic systems required investment in facilities and topdressing costs, the increase in output value brought by the substantial yield improvement fully offset the mentioned costs. The Gap between panels, with a 60.7% yield increase, could completely offset the yield loss caused by the land occupation of photovoltaic panels (about 20%). The two-year field experiment showed that the net income per hectare of the agrivoltaic agriculture mode increased by 1466 CNY, besides the land area occupied by photovoltaic panel construction, an increase of 18.1% compared with the conventional control outside the panels.

## 4. Discussion

### 4.1. Water–Fertilizer Coupling in Agrivoltaic Gaps Boosts Root Growth and Yield by Pod and Grain Number Enhancement

The farmland in the study area is degraded cropland, characterized by poor soil nutrient status, low organic matter content (4.46 g kg^−1^), frequent drought, and wind erosion. Crop production has long been jointly constrained by water deficit, nutrient deficiency, and wind–sand disturbance, resulting in low and unstable crop yield. This study showed that establishing a photovoltaic system combined with integrated water–fertilizer management in this region significantly improved soybean yield and its yield components. This yield-increasing effect resulted from the combined effects of photovoltaic array-induced microenvironmental improvement and precise water–fertilizer supply.

The agrivoltaic system regulated the stress environment experienced by crops through the installation of photovoltaic facilities and water–fertilizer management. The Open area still faced combined stresses such as wind–sand disturbance, drought, and soil infertility, whereas the photovoltaic areas significantly alleviated water and wind–sand stresses while maintaining relatively sufficient light availability. The photovoltaic panels acted as artificial wind barriers, which effectively reduced the mechanical damage to leaves from wind and sand, thus maintaining the stability of photosynthetic structures [23,24] and guaranteeing efficient dry matter accumulation. In addition, integrated water–fertilizer management ensured the efficient supply of water and nutrients during key growth stages, significantly alleviating the constraints of water deficiency and nutrient deficiency on soybean growth in degraded cropland. Compared with the Open treatment, soil organic matter, total nutrients, and available nutrients within the photovoltaic array were increased, providing a material basis for root growth, leaf expansion, reproductive organ development, and grain filling.

Water–fertilizer synergy promoted a significant increase in root length and root surface area, and enhanced water and fertilizer absorption, thus facilitating further photosynthate accumulation. It increased pod and grain number, and ultimately contributed to high yield in the Gap. The present study showed that the pod number and grain number per plant in the Gap were significantly higher than those in the Open treatment, with a yield increase of 60.7% on average. The crop in the Gap reached the highest HI among the treatments, which accords with the high-yield characteristics of strong source–large sink and efficient translocation [25,26]. These results were consistent with previous reports that crop yields and land use efficiency could be enhanced in the Gap with sufficient light by microclimate improvement in agrivoltaics system [18,27,28]. Therefore, the results of this study emphasize that, in water-limited regions, the combination of photovoltaic systems and integrated water–fertilizer management can improve crop productivity by alleviating water stress and creating a more balanced light–water–fertilizer resource environment.

The present study further verifies that in the Horqin Sandy Land, light is not a limiting factor, while water availability is more important, which is consistent with the regional differentiation mechanism reported by Jia et al. [18]. It reported that agrivoltaics could offer greater synergistic benefits across food, energy, water, and economics in the semi-arid area compared to those more humid eastern regions. Other recent global photovoltaic studies have shown that photovoltaic systems can improve soil moisture, reduce wind erosion, and stabilize the microenvironment in arid and semi-arid regions [13,14]. This study provides experimental evidence from degraded cropland in the Horqin Sandy Land for these findings, indicating that photovoltaic systems combined with integrated water–fertilizer management are an effective approach for improving the productivity and stability of degraded semi-arid cropland.

### 4.2. Microenvironmental Heterogeneity Within Photovoltaic Arrays Induces Distinct Crop Growth Adaptation Strategies

Within the photovoltaic array, the differences between the Gap and Under areas mainly reflected variations in radiation, water conditions, and crop physiological adaptation caused by the spatial position of photovoltaic panels. The Gap represented an optimized environment with coordinated light and water conditions, whereas the Under area represented a trade-off environment with sufficient water but insufficient light. This microenvironmental heterogeneity induced different growth and resource allocation strategies in soybean.

In the Gap area, soybean exhibited an active acquisition strategy: the aboveground parts accumulated assimilates through stronger photosynthesis, while the belowground parts expanded the range of resource uptake through a more developed root system. These two processes promoted each other and ultimately supported higher biomass and grain yield. In contrast, soybean in the Under area tended to adopt a conservative maintenance strategy. Shading by photovoltaic panels reduced the available light within the canopy, leading to decreased photosynthetic rate and limited accumulation of photosynthates. Meanwhile, soybean increased plant height and expanded leaf area to improve light capture capacity, and this shade-adaptive response helped plants utilize limited light resources as much as possible under low-light conditions [29,30].

Changes in root traits further indicated an adjustment of resource allocation strategies in the Under area. Root length, root surface area, and root tip number were markedly inhibited in the Under treatment, probably due to insufficient carbon supply to roots under low PAR conditions. When photosynthetic carbon acquisition is limited by shading, plants usually reduce investment in fine-root elongation and branching, and preferentially allocate limited assimilates to aboveground elongation, leaf expansion, and reproductive development [31,32]. On the other hand, the relatively favorable soil moisture and nutrient conditions under the panels also reduced the need for plants to acquire resources through deeper root penetration and the construction of a large fine-root system. Therefore, inhibited root growth accompanied by increased leaf area reflected a root–shoot trade-off under light limitation beneath the panels. This strategy may help plants preferentially expand the light-intercepting area, reduce belowground carbon consumption, and maintain reproductive growth to some extent in low-light environments [33]. In addition, the increased root diameter in the Under treatment indicated that roots under shading conditions tended to become thicker rather than elongating or forming more branches. This may suggest a shift in root strategy from extensive soil exploration toward maintaining transport and storage functions [34]. The increase in root diameter may also reflect reduced formation of fine absorptive roots and a higher proportion of structural tissues, which is a common root response under carbon allocation limitation [35]. Therefore, root thickening in the Under area should not be simply interpreted as improved root development, but rather as an adaptive adjustment to a low-light and high-moisture environment.

Due to the limited light environment beneath the panels, crops developed a root–shoot trade-off strategy characterized by increased leaf area, root thickening, and reduced fine-root proliferation, which enhanced the interception of limited light resources, reduced belowground carbon consumption, and maintained basic water transport and reproductive growth. However, morphological compensation could not fully offset insufficient incident radiation when shading was too strong, ultimately limiting photosynthate formation [36,37].

## 5. Limitation

Although this study provides field evidence that agrivoltaic systems can improve soybean performance under semi-arid sandy conditions, several limitations should be acknowledged. The specific limitations of this study are as follows: (1) The experiment was conducted for only two growing seasons at a single site in the southern Horqin Sandy Land; therefore, it could not fully represent interannual climatic variability and spatial heterogeneity across different semi-arid sandy regions. (2) The differences between the agrivoltaic treatments and the open-field conventional control treatment included not only photovoltaic shading but also differences in water–fertilizer management, because the Open treatment was not equipped with an integrated fertigation and irrigation system. The lack of an irrigated and fertilized open-field control limits the ability to fully distinguish the effects of photovoltaic-induced microenvironmental modification from those of improved field management. (3) Only one shade-tolerant soybean cultivar, Liaohei No. 5, was tested in this study, and the responses of other soybean cultivars or crop species may differ. (4) Although changes in soil nutrients were measured, soil microbial communities and biological processes were not analysed, which limits the interpretation of nutrient transformation mechanisms under photovoltaic arrays. (5) In this study, the local photovoltaic infrastructure was constructed as a public-welfare initiative by the government. Therefore, the economic assessment mainly evaluated the benefits from the perspective of agricultural profitability. Full photovoltaic infrastructure cost, maintenance, land occupation, depreciation period, and potential electricity revenue should also be taken into account in the calculation. (6) The water-saving and microclimate-regulating effects of the agrivoltaic system in this study were mainly inferred from soil water content and leaf gas-exchange parameters; evapotranspiration was not directly quantified, and microclimatic data, including air/canopy temperature, soil temperature, relative humidity, wind speed, and radiation dynamics were not continuously monitored. Therefore, multi-site, multi-year, and management-balanced experiments are still needed before broader technical recommendations can be made.

## 6. Conclusions

Photovoltaic systems combined with integrated water–fertilizer management improved soybean yield, harvest index, and soil water and nutrient conditions. In the Gap area, relatively sufficient light availability together with improved water and fertilizer conditions promoted root development, pod formation, and grain set, making this area the main contributor to yield improvement. Although the Under area was limited by shading, it developed adaptive responses such as reduced transpiration and morphological adjustment, including increased plant height and leaf area. This study provides preliminary field evidence that agrivoltaic systems can enhance crop productivity and improve soil conditions in degraded semi-arid cropland.

## Figures and Tables

**Table 1:** Analysis of variance for the effects of treatment (T), year (Y), and their interactions on plant height, leaf area, photosynthesis, root characteristics of soybean, and the contents of soil water and nutrients.

Source	*p*–Value																				
	Plant Height	Leaf Area	PAR	Pn	Gs	Ci	Tr	Leaf WUE	Root Length	Root Surface Area	Root Diameter	Root Tips	Soil Water Content	pH	SOC	TN	AN	TP	Olsen-P	TK	AK
T	**0.00**	**0.00**	**0.00**	**0.00**	**0.00**	**0.00**	**0.00**	**0.00**	**0.00**	**0.00**	**0.00**	**0.00**	**0.00**	**0.00**	**0.00**	**0.00**	**0.00**	**0.00**	**0.00**	**0.00**	**0.02**
Y	**0.03**	**0.03**	0.88	**0.04**	0.73	0.11	**0.03**	**0.00**	**0.00**	0.72	**0.00**	**0.01**	0.33	**0.03**	**0.00**	**0.00**	**0.00**	**0.00**	**0.00**	**0.00**	**0.00**
T × Y	**0.00**	0.73	0.41	0.32	**0.00**	**0.00**	0.10	**0.00**	**0.00**	0.37	**0.00**	0.83	0.67	**0.02**	0.56	0.61	**0.00**	0.22	0.21	**0.00**	0.96

Notes: The *p*–values less than 0.05 were highlighted in bold type.

**Table 2:** Effects of agrivoltaics on soybean yield and yield components during 2024 and 2025.

Treatment	Year	Pod Number per Plant	Number of Grains per Plant	100-Seed Weight (g)	Straw Yield (kg ha^–1^)	Grain Yield (kg ha^–1^)	Harvest Index
Open	2024	* 18.7 ± 2.08	39.7 ± 2.08	19.9 ± 0.85	2936 ± 112	3343 ± 136	53.2 ± 1.95
	2025	25.7 ± 5.69	54.7 ± 5.13	22.6 ± 0.97	3325 ± 92.3	3548 ± 404	51.6 ± 3.18
	2024~2025	22.2 ± 5.42 b	47.2 ± 8.93 b	21.3 ± 1.69 b	3131 ± 232 c	3445 ± 292 c	52.4 ± 2.54 a
Gap	2024	39.3 ± 1.53	92.7 ± 6.03	23.7 ± 0.26	4116 ± 225	5307 ± 222	56.3 ± 2.33
	2025	42.7 ± 2.89	93.3 ± 3.79	23.7 ± 0.22	4403 ± 499	5765 ± 349	56.7 ± 4.24
	2024~2025	41.0 ± 2.76 a	93.0 ± 4.52 a	23.7 ± 0.22 a	4259 ± 380 a	5536 ± 362 a	56.5 ± 3.07 a
Under	2024	35.7 ± 3.06	84.3 ± 3.06	22.8 ± 0.83	3632 ± 148	4496 ± 163	55.3 ± 1.62
	2025	40.7 ± 2.08	89.0 ± 6.00	23.6 ± 0.28	3908 ± 278	5027 ± 252	56.3 ± 2.47
	2024~2025	38.2 ± 3.60 a	86.7 ± 4.97 a	23.2 ± 0.70 a	3770 ± 250 b	4762 ± 347 b	55.8 ± 1.94 a
P	Treatment	0.000	0.000	0.000	0.000	0.000	0.050
	Year	0.005	0.009	0.003	0.026	0.009	0.927
	Treatment × Year	0.620	0.051	0.011	0.920	0.569	0.683

Notes: The different letters indicate a significant difference among treatments (*p* < 0.05). Open, open-field conventional control; Gap, between the panels; Under, under the panels. * Mean value ± standard error (*n* = 18).

**Table 3:** Economic benefits of the agrivoltaics during 2024 and 2025.

Year	Treatments	Mechanical Operation (CNY ha^–1^)	Seeds (CNY ha^–1^)	Fertilizers (CNY ha^–1^)	Cost of Water and Fertilizer Integration Facilities (CNY ha^–1^)	Fertilizer Application by Irrigation (CNY ha^–1^)	Labor Cost (CNY ha^–1^)	Yield (kg ha^–1^)	Price (Yuan kg^–1^)	Net Revenue (CNY ha^–1^)
2024	Conventional	1350	540	1620	0	0	600	3343	3.6	7924
	Agrivoltaics	1800	540	1215	225	1200	600	4083	3.6	9119
2025	Conventional	1350	540	1620	0	0	600	3548	3.5	8308
	Agrivoltaics	1800	540	1215	225	1200	600	4464	3.5	10,044

Notes: The yield of the agrivoltaics refers to the average yield of the Gap and Under (in the photovoltaic system, the area of crop cultivation accounts for 80%, in which the Under was 20%, and the Gap was 60%; the remaining 20% land area is occupied by panel infrastructure). Net income = total output value − sum of various costs.

## Data Availability

The data presented in this study are available on request from the corresponding author due to privacy.
